# Interleukin-10-592 polymorphism: impact on relapse and survival after allogeneic hematopoietic stem cell transplantation in children with hematological malignancies

**DOI:** 10.1007/s00432-021-03695-3

**Published:** 2021-06-21

**Authors:** Laura Schwenk, Susan Wittig, Bernd Gruhn

**Affiliations:** Department of Pediatrics, Jena University Hospital, Am Klinikum 1, 07747 Jena, Germany

**Keywords:** Interleukin 10, Single nucleotide polymorphism, Allogeneic hematopoietic stem cell transplantation, Children, Relapse rate, Event-free survival

## Abstract

**Purpose:**

Interleukin-10 (IL-10) potentially can promote the development of alloimmunity. The aim of this study was to investigate if the IL-10-592 CC genotype in the donor reduces the risk of relapse after hematopoietic stem cell transplantation (HSCT) and if that has an impact on event-free survival (EFS) and overall survival (OS).

**Methods:**

A cohort of 211 children with acute lymphoblastic leukemia (*n* = 99), acute myeloid leukemia (*n* = 69), myelodysplastic syndrome (*n* = 31) or chronic myeloid leukemia (*n* = 12) who underwent hematopoietic stem cell transplantation (HSCT) in a single center and their respective donors were genotyped of IL-10 gene for rs1800872 using TaqMan real-time polymerase chain reaction.

**Results:**

The IL-10-592 CC genotype was detected in 107 of the 211 donors (50.7%) and in 106 of the 211 patients (50.2%). Genotype AC was found in 95 donors (45.0%) and in 90 patients (42.7%). Nine donors (4.3%) and 15 patients (7.1%) were homozygous for AA. Ultimately, we observed a significantly reduced incidence of relapse rate (RR) in patients who were transplanted from a donor with the IL-10-592 CC genotype (19% versus 43% (AC) versus 49% (AA); *P* = 0.0007). In addition, a significant increase of EFS (*P* = 0.004) and OS (*P* = 0.006) was detected if the IL-10-592 CC genotype is present in the donor. The occurrence of the IL-10-592 CC genotype, in either donors or recipients, had no significant impact on acute and chronic graft-versus-host disease. In addition, the IL-10-592 genotype of the recipients was not relevant for the RR (*P* = 0.47434), the EFS (*P* = 0.840), and the OS (*P* = 0.535).

**Conclusion:**

The IL-10-592 CC genotype in the donor was associated with a significant decrease of RR which led to a significant increase of EFS and OS after HSCT. This is the first study to describe an association of the IL-10 gene polymorphism with RR, EFS, and OS after HSCT. Selecting a donor with the IL-10-592 CC genotype could be a useful therapeutic strategy for improving the outcome after allogeneic HSCT.

## Introduction

Leukemia is the most common malignant disease in childhood. The majority of patients with leukemia are cured by means of chemotherapy. High-risk patients with a greater risk of relapse or patients with high-risk relapse require a more intensive therapy. The allogeneic hematopoietic stem cell transplant (HSCT) is an efficient strategy to treat those patients. However, this treatment also includes various risks, such as graft-versus-host disease (GVHD), infections, hepatic sinusoidal obstruction syndrome, and relapse (Hierlmeier et al. 2018; Dukat-Mazurek et al. 2017). To avoid those complications an appropriate donor is desirable (Lin et al. 2003). Ideally, the donor should transmit a graft-versus-leukemia (GVL) effect, but should not induce a GVHD (Seggewiss and Einsele 2010). Interleukin-10 (IL-10) is an antiphlogistic homodimer, consisting of 160 amino acids, belonging to the group of the cytokines (Wei et al. 2019). In 1989, after experiments with laboratory mice, IL-10 was described as a cytokine synthesis inhibiting factor produced by type 2T-helper cells (Fiorentino et al. 1989). Furthermore, IL-10 inhibits the secretion of cytokines of type 1T-helper cells (Fiorentino et al. 2016). In addition to the type 2T-helper cells, IL-10 is also produced by multiple other cell types, including monocytes, B cells, dendritic cells (Sabat et al. 2010), keratinocytes (Enk and Katz 1992), and tumor cells (Asadullah et al. 2000).

The human IL-10 gene is located on chromosome 1q31-q32, consists of five exons and has a molecular weight of 18.5 kDa (Zhang and Kuchroo 2019). The effect of IL-10 is mediated by the binding to the IL-10 receptor which is a specific transmembrane receptor (Moore et al. 2001). The IL-10 receptor (IL-10R) is composed of two subunits, which both belong to the class 2 cytokine receptors, IL-10R1 (formed by hematopoietic stem cells) and IL-10R2 (expressed ubiquitously) (Walter 2014). Acuner-Ozbabacan et al. (2014) described IL-10 as a well-known immunomodulatory cytokine within the immune system. The characteristics of IL-10 are still disputed throughout medical publications, and IL-10 is described by numerous authors as a paradoxical cytokine (Mannino et al. 2015; Geginat et al. 2019; Wang et al. 2013). It is acknowledged and scientifically proven that IL-10 has immunosuppressive effects by inhibiting the production of various cytokines (IL-1α, IL-1β, IL-6, IL-10 itself, TNF, GM-CSF, G-CSF, M-CSF, LIF, PAF), CC chemokines (MCP-1, MCP-5, CCL5) and CXC chemokines (IL-8, IP-10, MIP-2) by monocytes and macrophages (de Vries 1995; Zheng et al. 2020). In addition, IL-10 influences the expression of MHC class II molecules and CD86/CD80 in antigen-presenting cells (Mittal and Roche 2015), the expression of MHC class I molecules in tumor cells (Salazar-Onfray et al. 1997), the expression of costimulatory molecules on dendritic cells and regulates the IL-12 production (Rahim et al. 2005). IL-10 has an immunosuppressive effect, yet, contradicting this statement, in the context of malignant diseases there are numerous investigations on in vitro and in vivo laboratory mice which prove the immune support of the IL-10’s function (Zheng et al. 1996; Mocellin et al. 2005). These investigations demonstrate that IL-10 stimulates an anti-tumor cytotoxic reaction and therefore a tumor regression leading to the prevention of metastases formation. These results also include the ability of IL-10 to increase the cell count of CD8 + T cells, the increase of interferon gamma’s secretion and the induction of cytotoxicity in tumors (Lauw et al. 2000). Furthermore IL-10 was able to decrease the growth of tumors and to generate the rejection of the tumors (Berman et al. 1996).

As far as we know, for the first time, we analyzed the influence of IL-10 polymorphism on the relapse rate (RR), the event-free survival (EFS), and the overall survival (OS) after allogeneic HSCT. We were particularly interested in examining the impact of IL-10 single nucleotide polymorphism (SNP) rs1800872 as a possible factor for the decrease of RR after HSCT.

## Patients and methods

### Patients

We analyzed 211 patients together with their donors at the Department of Pediatrics, Jena University Hospital, Jena, Germany. The patients suffered from the following diseases: acute myeloid leukemia (AML), acute lymphoblastic leukemia (ALL), chronic myeloid leukemia (CML) and myelodysplastic syndrome (MDS). The patients’ characteristics are shown in Table 1.

**Table 1:** Characteristics of patients and donors (n = 211)

Characteristics	Total, No (%)
Median age of the patients (y)	11
Age range	5 months—18 years
Sex of the patients	
Male	123 (58.3)
Female	88 (41.7)
Disease	
Acute lymphoblastic leukemia	99 (46.9)
Acute myeloid leukemia	69 (32.7)
Chronic myeloid leukemia	12 (14.7)
Myelodysplastic syndrome	31 (14.7)
Remission (complete)	
First	65 (30.8)
Second	42 (19.9)
Third	14 (6.6)
Not in remission	90 (42.7)
Conditioning regimen (based on)	
Total body irradiation	107 (50.7)
Busulfan	104 (49.3)
Donor type	
HLA-matched unrelated	108 (51.2)
HLA-mismatched unrelated	26 (12.3)
HLA-identical related	61 (28.9)
HLA-haploidentical related	16 (7.6)
GvHD	
aGvHD II-IV	74 (35.1)
aGvHD III-IV	30 (14.2)
cGvHD	36 (17.1)
Stem cell source	
Bone marrow	121 (57.3)
Peripheral blood stem cells	90 (42.7)
Donors’ IL-10 polymorphism distribution	
CC	107 (50.7)
AC	95 (45.0)
AA	9 (4.3)

### Genotyping of IL-10 polymorphism

For the genotyping of IL-10 polymorphism DNA was isolated from the patients’ blood or bone marrow aspirates using High Pure PCR Template Preparation Kit (Roche, Mannheim, Germany) according to the manufacturer’s instructions. The DNA concentration was measured at 260 and 280 nm using BioPhotometer plus provided by Eppendorf (Wesseling-Berzdorf, Germany). The mixture consisted of 1 µL (10 ng/µL) DNA, 10 μL Genotyping Mastermix, 9.5 μL sterile aqua, and 0.5 μL primer–probe mix (TaqMan Genotyping Assays provided by Applied Biosystems, Foster City, CA, USA). We transferred at least five negative controls together with the samples using pipettes into 96-well optical reaction plates marked with barcodes. We used the 7900HT Fast Real-Time PCR System by Applied Biosystems to subject the DNA to an absolute quantification process by proceeding with the following: heating of the samples for 10 min at 95 °C to activate the mixture immediately followed by 40 cycles of 15 s duration at 92 °C to denaturize the DNA and finishing with 60 s at 60 °C to anneal and extend the DNA. Subsequently, the SNP was analyzed through an allelic discrimination post-read run. The investigated SNP in this process was rs1800872 (IL-10-592).

### Statistical analysis

We aimed to find a potential association between the analyzed IL-10-592 polymorphism and the RR, EFS, and OS. We used the Kaplan-Maier method for providing maximum accuracy concerning the calculations of the EFS and OS. With the help of the log-rank test we were able to identify all differences between results. OS was defined as the duration between HSCT and the death of the patient. Subsequently, EFS was defined as the duration between HSCT and any of the following events: relapse, secondary malignancy, or death. For the analysis of the RR, survival calculations with competing risk were used. The Gray test (Gray 1988) was used for evaluating differences between the statistical curves. RR was defined as the cumulative incidence of relapse. For all calculations, IBM SPSS Statistics 26 and R Foundation for Statistical Computing 4.0.2. were used. The *P* value below 0.05 was considered as statistically significant. We performed multivariate analyses for the identification of potential confounding variables such as gender match, disease risk, and acute graft-versus-host disease grade (GVHD) II–IV. The disease risk was low if the patients were transplanted in complete first or second remission. The disease risk was high if the transplantation was performed in more than second remission or in relapse (Arndt et al. 2014).

## Results

### Allocation of polymorphism

In total, we included 211 donors and 211 recipients in our examinations. While analyzing the information of all donors, we observed the homozygous genotype CC in 107 donors (50.7%), the heterozygous genotype AC in 95 donors (45.0%), and the homozygous genotype AA in 9 donors (4.3%). The IL-10-592 CC genotype was detected in 106 of the 211 recipients (50.2%). Genotype AC was found in 90 patients (42.7%) and 15 patients (7.1%) were homozygous for AA.

### Relapse rate (RR)

**Fig. 1:**
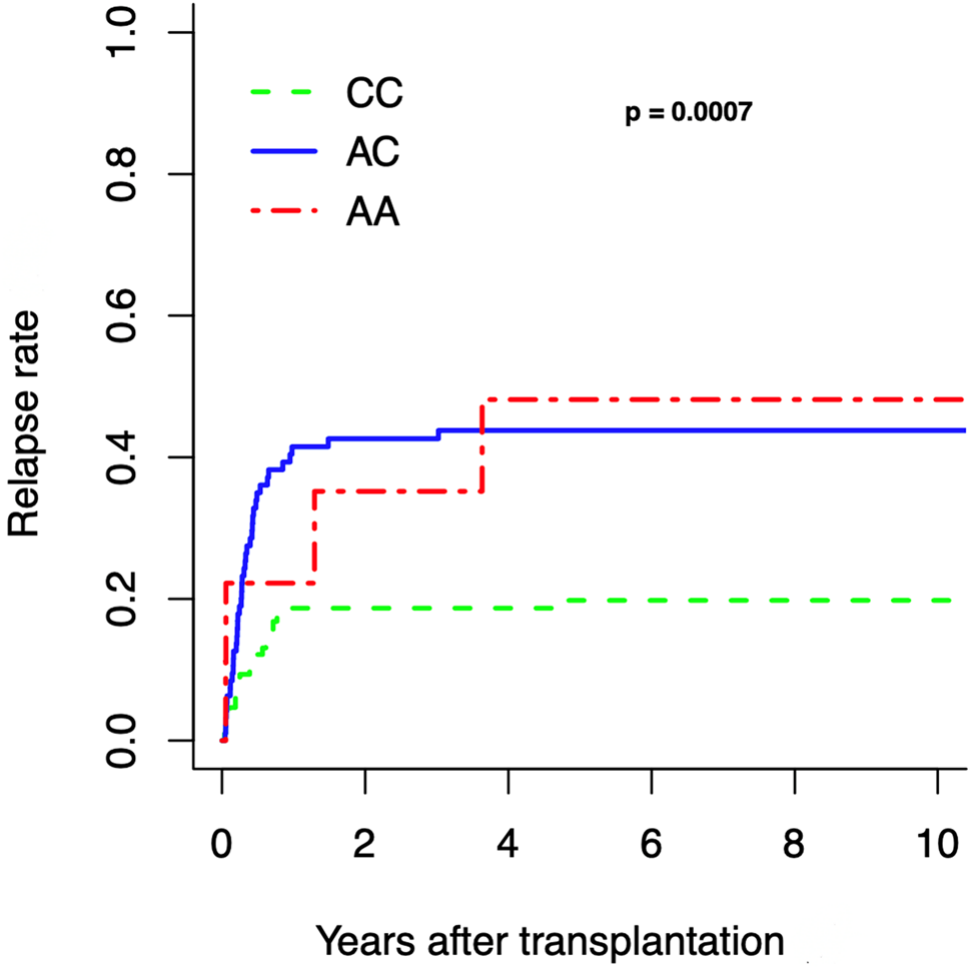
Relapse rate according to donor’s SNP rs1800872 status

The analysis of the donors’ IL-10-592 SNP showed that the genotype CC was associated with a decreased RR. Only 19% of the patients who received a transplant from a donor with genotype CC suffered from relapse compared to 43% or 49% of patients who received a transplant from a donor with genotype AC or AA, respectively (*P* = 0.0007; Fig. 1).

### Event-free-survival (EFS)

**Fig. 2:**
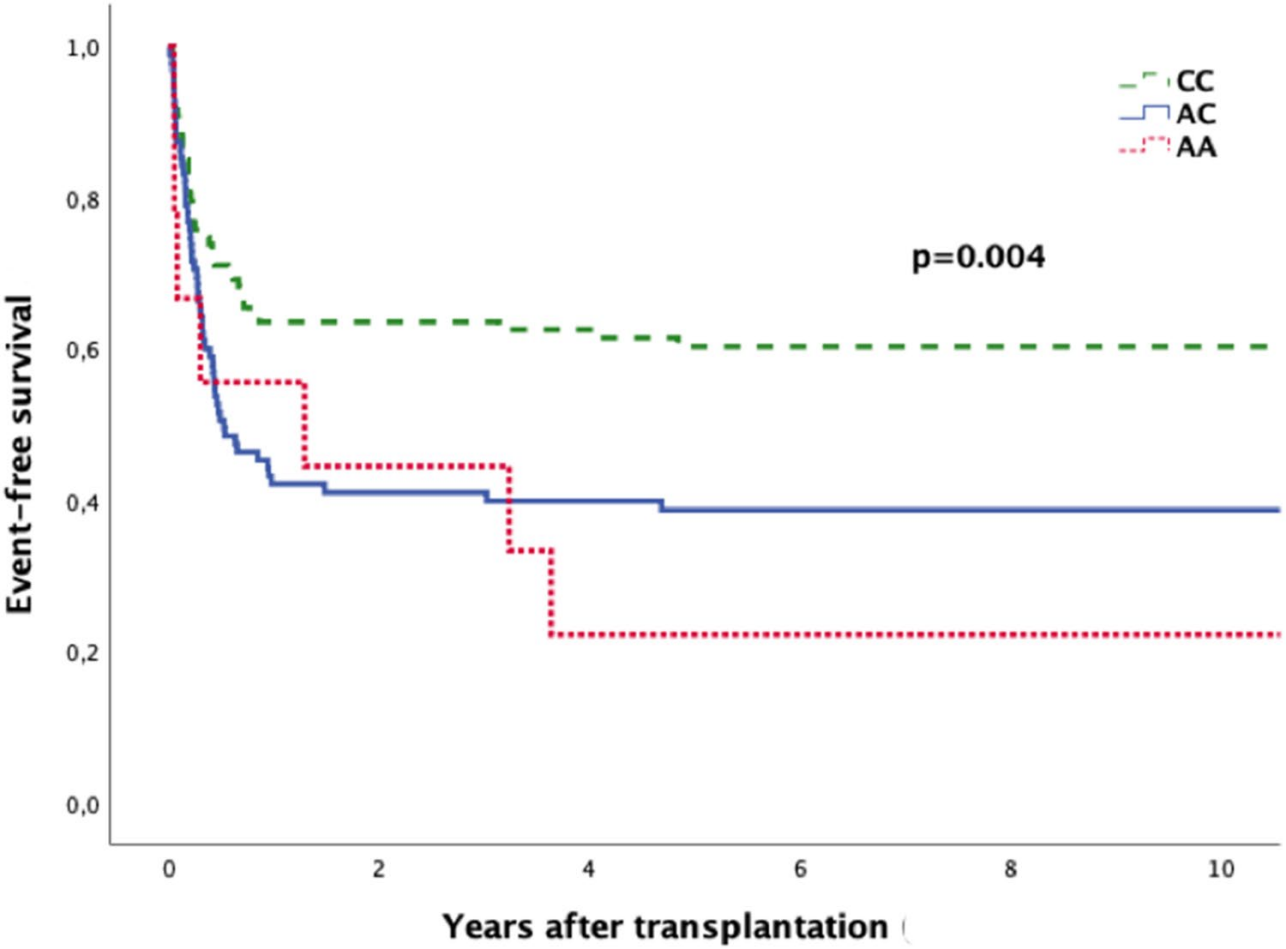
Event-free survival according to donor’s SNP rs1800872 status

We observed a significant association between the genotype of the donor and the EFS. The EFS was 60.7% if the patient was transplanted from a donor with the genotype CC. On the other hand, the EFS was only 38.9% or 22.2% if the patient was transplanted from a donor with the genotype AC or AA, respectively (*P* = 0.004; Fig. 2).

### Overall survival (OS)

**Fig. 3:**
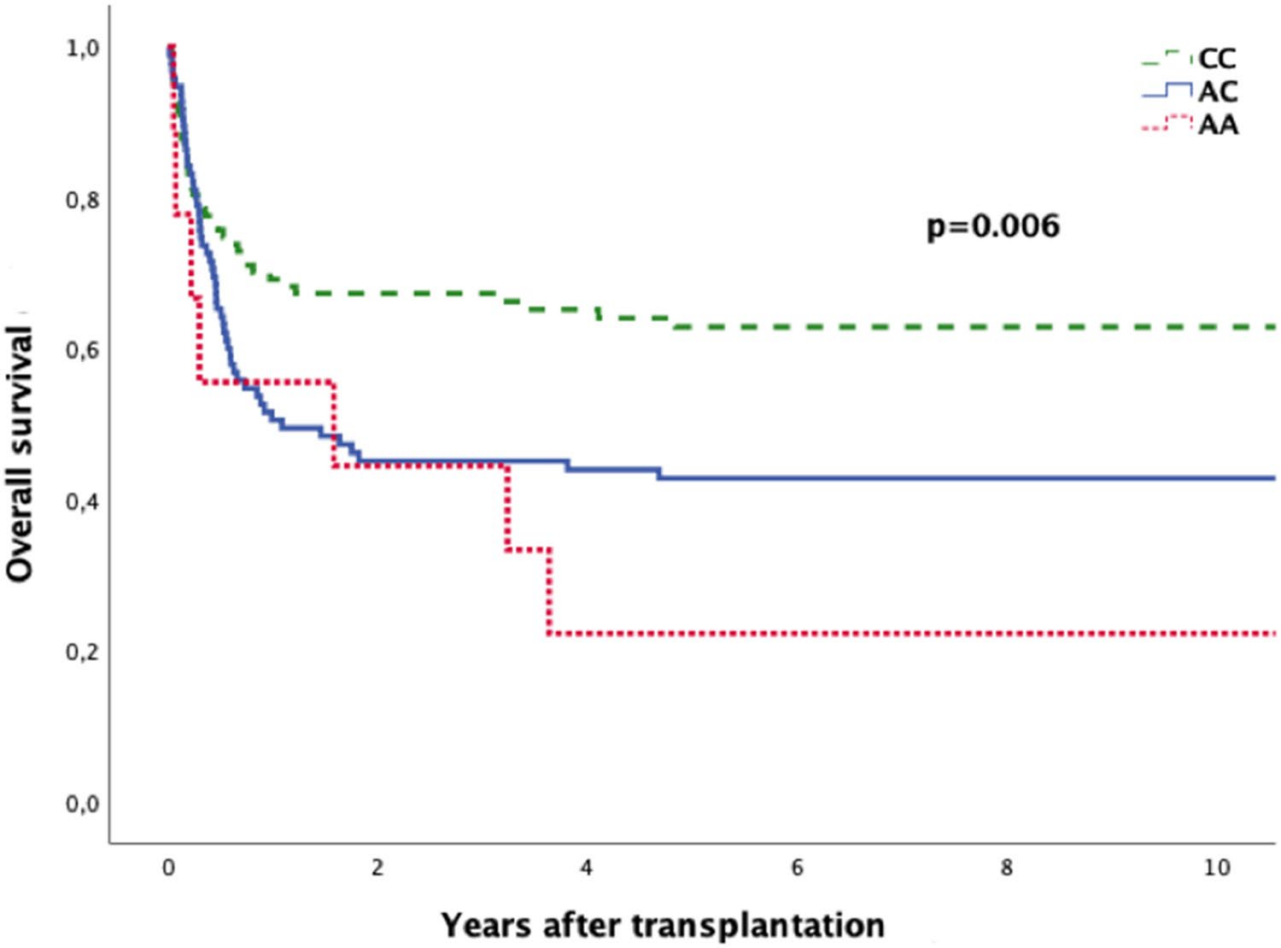
Overall survival according to donor’s SNP rs1800872 status

We found a significant association between the donors’ genotype and the OS. The OS was 63.3, 43.2, or 22.2% if the patient was transplanted from a donor with the IL-10-592 genotype CC, AC or AA, respectively (*P* = 0.006; Fig. 3). In addition, the IL-10-592 genotype of the recipients was not relevant for the RR (*P* = 0.47434), the EFS (*P* = 0.840), and the OS (*P* = 0.535).

### Multivariate analysis

Multivariate analysis was used to prove whether the association between the donor’s IL-10-592 polymorphism and RR and EFS was influenced by other factors. To assess whether IL-10-592 SNP is an independent prognostic factor, we incorporated the following possible confounding variables: donor-recipient-gender match, disease risk, stem cell source, conditioning regimen, HLA compatibility, and the occurrence of acute GVHD grade II to IV. The results are presented in Table 2 and demonstrate that the IL-10-592 SNP is an independent significant risk factor for RR and EFS.

**Table 2:** Multivariate analysis. Relapse rate (RR) and event-free survival (EFS)

Variable	RR		EFS	
	HR (95% CI)	*P*	HR (95% CI)	*P*
Donor IL10- (rs1800872)	0.438 (0.241–0.797)	0.007	0.592 (0.396–0.886)	0.011
Gender match	1.464 (0.844–2.538)	0.175	1.447(0.962–2.176)	0.076
Disease risk	0.001 (0.0–1.321)	0.760	0.255 (0.167–0.391)	0.001
Stem cell source	1.494 (0.871–2.562)	0.144	1.526 (1.029–2.265)	0.036
Conditioning regimen	0.994 (0.599–1.650)	0.982	0.856 (0.579–1.264)	0.433
HLA compatibility	1.226 (0.438–3.432)	0.698	0.515 (0.231–1.152)	0.106
Acute GVHD grade II-IV	1.504 (0.864–2.617)	0.149	1.038 (0.693–1.554)	0.858

*GVHD* graft-versus-host disease, *HLA* human leukocyte antigen

## Discussion

This retrospective study demonstrates that IL-10-592 polymorphism CC of the donor had a significant impact on the RR (*P* = 0.0007), the EFS (*P* = 0.004) and the OS (*P* = 0.006).

Additionally, we examined the impact of the IL-10-592 polymorphism CC on the acute and chronic GVHD. However, we did not observe a significant association between the values. In our study, we found that the genotype CC provides a strong protection against relapse leading to a significant increase of EFS and OS. IL-10 is a central immunomodulatory cytokine. Previous studies have demonstrated that IL-10 leads to the formation of cytotoxic T lymphocytes and activation of B cells by upregulation of specific genes in toll-like-receptor-activated macrophages and dendritic cells (Lang et al. 2002; Iyer and Cheng 2012).

The immunostimulatory effects of IL-10 were also examined by other authors in malignant diseases (Suzuki et al. 1995; Mosser and Zhang 2008). IL-10 stimulation promotes a T-cell activation and the differentiation of CD8 + T cells (Mannino et al. 2015). This activation of T cells could be the explanation of the reduced RR which we have found in our study. After the treatment with IL-10, patients showed a significant decrease of interleukin-1 (IL-1) and increased interferon-gamma levels (Tilg et al. 2002; Lauw et al. 2000). Until now, IL-1 has been known as a proangiogenic cytokine and promoter of tumor formation (Voronov et al. 2007). The transfection of tumor cells with IL-10 or another systemic therapy of IL-10 suppressed the tumor growth and caused tumor rejection. In addition, the genetic ablation of IL-10 in the mouse increased the sensitivity to chemical carcinogenesis, augmented the growth of transplanted tumors and accelerated the formation of metastases (Berti and Brajão de Oliveira 2018). The tumor-microenvironment consists of regulatory T cells, dysfunctional antigen-presenting cells and myeloid-derived suppressor cells. Exactly those cells create an immunosuppressive network and can therefore promote tumor growth. We want to highlight that the cytokine IL-10 hampers the development of previously mentioned cells.

In conclusion, IL-10 is responsible for inhibiting tumor development, tumor growth and formation of metastases. Furthermore, IL-10 can regulate inflammatory cytokine production. Aborsangaya et al. (2007) has demonstrated that the IL-10-592 CC genotype causes a higher production rate of IL-10 than the CA- or AA-genotype. These observations agree with our results that the genotype IL-10-592 CC in the donor induced a significant decrease of RR as well as a significant increase of EFS and OS after HSCT in children with hematological malignancies. To our knowledge, this is the first study to describe an association of IL-10 gene polymorphism with RR after HSCT. Selecting a donor with the IL-10-592 CC genotype could be useful therapeutic strategy for improving the outcome after allogeneic HSCT. Additionally, further studies are necessary to confirm our results.
